# Factors affecting the value of diffusion-weighted imaging for identifying breast cancer patients with pathological complete response on neoadjuvant systemic therapy: a systematic review

**DOI:** 10.1186/s13244-021-01123-1

**Published:** 2021-12-18

**Authors:** Kay J. J. van der Hoogt, Robert J. Schipper, Gonneke A. Winter-Warnars, Leon C. ter Beek, Claudette E. Loo, Ritse M. Mann, Regina G. H. Beets-Tan

**Affiliations:** 1 Department of Radiology, The Netherlands Cancer Institute – Antoni van Leeuwenhoek Hospital, Plesmanlaan 121, 1066 CX Amsterdam, The Netherlands; 2 GROW School of Oncology and Developmental Biology, Maastricht University Medical Center, Maastricht, The Netherlands; 3 Department of Medical Physics, The Netherlands Cancer Institute – Antoni van Leeuwenhoek, Amsterdam, The Netherlands; 4 Department of Radiology and Nuclear Medicine, Radboud University Medical Center, Nijmegen, The Netherlands; 5 Danish Colorectal Cancer Unit South, Institute of Regional Health Research, Vejle University Hospital, University of Southern Denmark, Odense, Denmark

**Keywords:** Breast cancer, pCR, DWI, Neoadjuvant, Methodology

## Abstract

This review aims to identify factors causing heterogeneity in breast DWI–MRI and their impact on its value for identifying breast cancer patients with pathological complete response (pCR) on neoadjuvant systemic therapy (NST). A search was performed on PubMed until April 2020 for studies analyzing DWI for identifying breast cancer patients with pCR on NST. Technical and clinical study aspects were extracted and assessed for variability. Twenty studies representing 1455 patients/lesions were included. The studies differed with respect to study population, treatment type, DWI acquisition technique, post-processing (e.g., mono-exponential/intravoxel incoherent motion/stretched exponential modeling), and timing of follow-up studies. For the acquisition and generation of ADC-maps, various *b*-value combinations were used. Approaches for drawing regions of interest on longitudinal MRIs were highly variable. Biological variability due to various molecular subtypes was usually not taken into account. Moreover, definitions of pCR varied. The individual areas under the curve for the studies range from 0.50 to 0.92. However, overlapping ranges of mean/median ADC-values at pre- and/or during and/or post-NST were found for the pCR and non-pCR groups between studies. The technical, clinical, and epidemiological heterogeneity may be causal for the observed variability in the ability of DWI to predict pCR accurately. This makes implementation of DWI for pCR prediction and evaluation based on one absolute ADC threshold for all breast cancer types undesirable. Multidisciplinary consensus and appropriate clinical study design, taking biological and therapeutic variation into account, is required for obtaining standardized, reliable, and reproducible DWI measurements for pCR/non-pCR identification.

**Supplementary Information:**

The online version contains supplementary material available at 10.1186/s13244-021-01123-1.

## Key points

- Large heterogeneity/variability in studies hampers successful clinical implementation of DWI metrics.
- Technical variability was encountered in, e.g., *b*-value combinations, ROIs, and models.
- Clinical heterogeneity was observed (e.g., scan-moment during treatment, tumor type differentiation, and NST-protocol)
- Multi-disciplinary consensus/cooperation is required for proper clinical study design.
- Quality control and standardization are essential for clinical and technical validation.

## Introduction

Women with breast cancer are increasingly treated with neoadjuvant systemic therapy (NST) [1]. The optimal response is achieved when at subsequent surgical pathology no residual cancer is detected (pathological complete response, pCR). Between subtypes, pCR rates vary widely from 0.3% (luminal A) to 60% (HER2-type) [2].

To identify breast tumor pCR, a diagnostic lumpectomy is currently necessary, albeit for therapeutic reasons this may no longer be required. Identifying pCR with imaging only would be a significant improvement, as it would prevent needless surgical procedures. However, this requires that non-pCR is accurately detected. Only then omitting surgery can be accepted with a wait-and-see strategy as a practical and reliable alternative. Such an approach is already proposed for colorectal cancer treated with neo-adjuvant chemo-radiotherapy [3]. In the case of breast cancer, ^18^F-FDG PET-CT and/or dynamic contrast-enhanced (DCE) magnetic resonance imaging (MRI) is extensively investigated to predict and evaluate NST-response [4, 5]. Despite all these efforts, NST response assessment still needs to be improved. The percentage of correctly identified pCR on MRI appears too low to safely omit diagnostic lumpectomy [6]. Furthermore, the accuracy of DCE-MRI seems to depend on the cancer subtype [6, 7]. In addition, the potential risk of the observed gadolinium deposition in the deep nuclei of the brain after repeated exposure to gadolinium-based contrast agent has raised some concerns [8]. Therefore, other MRI-techniques, like diffusion-weighted imaging (DWI), are investigated [9].

While DCE provides information on perfusion, DWI provides information about cell density and tissue microstructure based on the diffusion of tissue water. Tumors with high cell density have a relative low apparent diffusion coefficient (ADC), which theoretically increases when the density is reduced by chemotherapy. However, this is not observed in all tumors, since ADC is dependent on multiple factors [10].

The use of DWI might be beneficial for the response assessment of NST, as microstructure changes may be detected at an earlier stage than tumor size reduction [11]. Previous reviews reported aggregate values on the performance of DWI–MRI for predicting or identifying pCR. Chu et al. reported a sensitivity = 0.88, and specificity = 0.79 [12]; similarly, Gao et al. reported sensitivity = 0.89 and a specificity = 0.72 [13]. However, reported cutoff ADC-values in the individual studies appear variable, preventing the use of a single cutoff value to achieve such performance. It is, therefore, uncertain whether these aggregate performance measures are valid. In addition, studies vary in including factors, such as patient selection, tumor subtypes, and NST-types. Moreover, the methodology used for quantitative analysis of DWI–MRI is not uniform. To partly solve this issue, Baltzer et al. published a EUSOBI consensus paper regarding DWI of the breast for lesion classification. However, the consensus paper does not provide insights on issues applicable in treatment monitoring using DWI for identifying patients with pCR [14]. To shed a light on the magnitude of these issues, this review aims to identify technical, clinical, and biological heterogeneity and their impact in DWI studies identifying pCR on NST. The final aim is to support a more robust implementation of quantitative DWI for NST monitoring in breast cancer patients.

## Materials and methods

### Search, inclusion/exclusion criteria, and quality assessment

A PubMed-search was performed until April 2020, using Medical Subject Headings (MeSH)- and free-text terms for breast cancer, NST, DCE, DWI, and pCR. Identified abstracts were read and selected by two researchers. Abstracts were excluded when they were: (1) not published in English; (2) not about human breast cancer; (3) studies that performed no prediction/evaluation of the breast tumor with pCR; (4) studies that did not compare outcome to histopathology; (5) studies with neoadjuvant therapy using radiotherapy; (6) comment on; (7) meta-analysis; (8) case report.

**Fig. 1:**
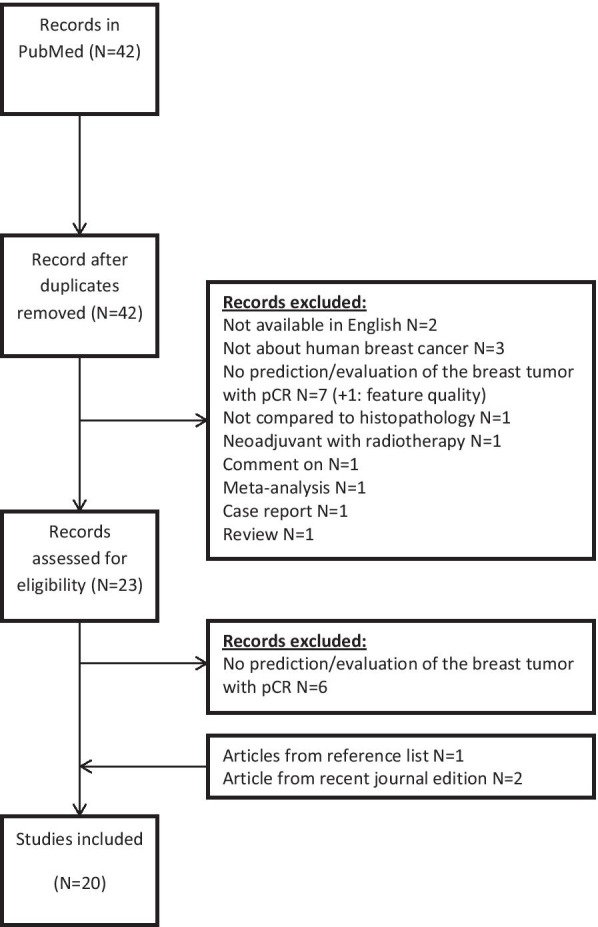
Flow chart selection process review

After selection, the references of included studies were checked for extra studies (selection process: Fig. 1). Finally, quality of included studies was assessed using QUADAS-2 [15].

### Data extraction and analysis

Data were extracted based on general parameters (e.g., first author, publication year), clinical characteristics (e.g., type of tumor, neoadjuvant treatment protocol), scan-moments (i.e., before, during (number of cycles) and/or post-NST), MRI/DWI protocol parameters (e.g., *B*_0_-field strength (T), *b*-values (s/mm^2^)), and details on the measures derived from the DWI data (e.g., ADC (mm^2^/s)). The reported performance measures per study were collected. For pCR prediction/detection, pCR-definitions were also extracted, since studies could permit different degrees of residual (tumor) tissue for pCR.

If performance measures were missing, reconstruction was tried by extracting data (from full-text/supplementary material) normally used in 2 × 2 contingency tables. In this review, pCR and non-pCR are defined as, respectively, positive and negative events.

After data extraction, grouping of results based on comparable study methodologies/definitions was performed. Data were analyzed by comparing study population (-related) and MR (-related) parameters to outcomes in terms of distinguishing pCR/non-pCR.

Sub-analyses were performed on different pCR-definitions (regarding in- or exclusion of residual ductal carcinoma in situ (DCIS)), when sufficient data were available.

Due to expected heterogeneity, we did not initially intend to conduct formal data-pooling and/or meta-analysis. Post hoc analysis of the results also prohibited this.

## Results

### Search strategy and study selection

The search (Additional file 1: Search term combinations in PubMed) resulted in 42 unique publications. After selection, 20 publications were included (Fig. 1). QUADAS-2 [15] assessment identified sources of bias and applicability concerns present in most studies (Table 1). In some studies, the patient selection might have initiated bias by using a non-representative study population (e.g., not describing the group as consecutive, small research populations in a large time interval, tumor diameter as exclusion criterion). Furthermore, several studies included patients who had a different number of scans within the study.

**Table 1:**
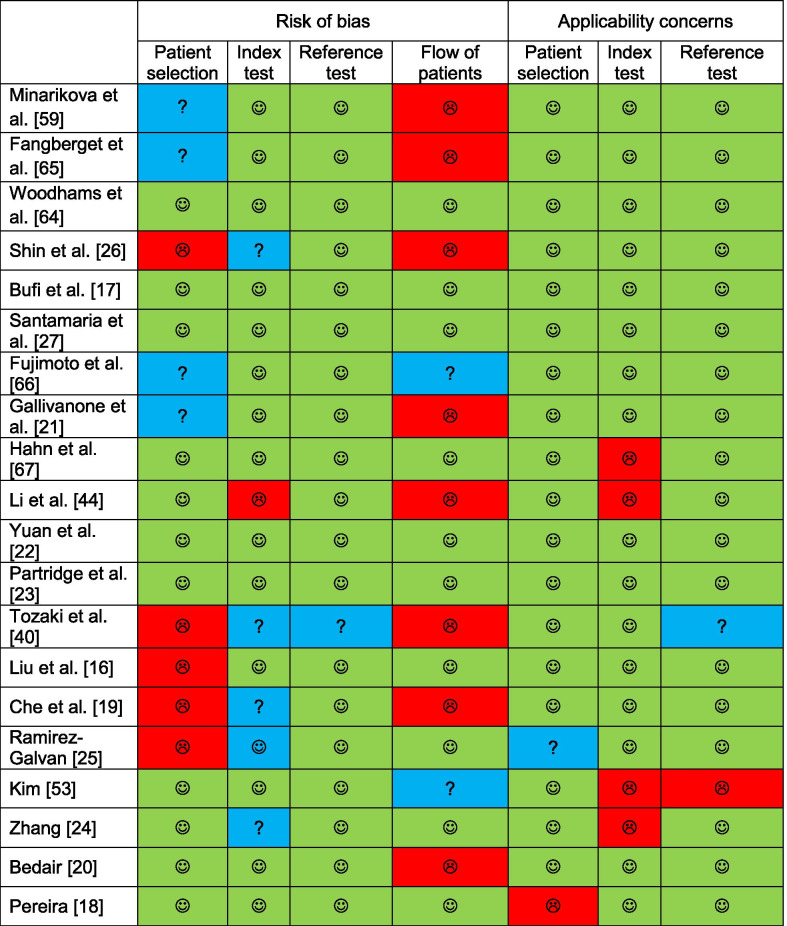
Risk of bias and applicability concerns of included studies

low risk,  high risk,  unclear risk

### General study characteristics

A general overview of the study characteristics is presented in Tables 2 and 3. In total, approximately 1455 patients/lesions were included (range per study: 7–242) (Table 2). Most studies were retrospective single center. NST-regimens varied widely between and within the studies. The pCR-ratios varied (12.9–39.3%), reflecting the variability and wide distribution of histological and molecular subtypes in the studies. In addition, the pCR-definitions differed between studies. Taking this into account, we noted that in general higher pCR-ratios were observed with less stringent pCR-definitions (especially for DCIS), as shown in Table 4.

**Table 2:** General study parameters

First author	Year	Study design	Age (y)	Patients (DWI)	Lesions	Initial stage	NST (cycles)	pCR rate (%)^a^	Molecular subtype*
Woodhams [64]	2010	Prospective/single center		69	70		CA-T^(4+4)^	12.9	
Tozaki [40]	2010	Prospective/single center	46 (27–61)	7	7	II	FEC 75^(1)^ or FEC 75^(4)^ or FEC 100^(4)^ or FEC 75^(2)^ or weekly Pac^(4)^	14.3	
Fangberget [65]	2011	Prospective/single center	50.7 (37–72)	Pre: 31 4 cy: 27	32		FEC^(4+2)^, FEC^(4)^-T(+trastuzumab if HER2+)	36.6^b^	TN: 4 ER+: 21 HER2 enriched: 5 ER/Pr−/Her2+: 1
Shin [26]	2012	Retrospective/single center		41			CA^(4)^, C-T^(4+4)^, TA, FEC	36.6	
Fujimoto [66]	2014	Retrospective/single center	50.9 ± 10.0 (29–70)	56		II and III	Different regimes^c^	14.3^d^	HER2+: 17 Hormone+: 40
Hahn [67]	2014	Retrospective/single center	43.3 (24–59)	1.5 T: 28 3.0 T: 50	78	II and III	Different combinations according to receptor status	24.4	ER+: 40/78 HER2+: 23/78
Bufi [17]	2015	Retrospective/single center	47 ± 10.1	225		II, III and IV	Combination of TAC (not specified)	17.3^e^	Luminal: 143 TN: 37 HER2+/enriched:: 17 Hybrid: 28
Li [44]	2015	Prospective/single center	Median 46 (28–67)	Start: 42 1 cy: 36		II and III	Different combinations	33.3	TN: 12 Hormone+: 19 HER enriched: 11
Liu [16]	2015	Retrospective/single center	53.2 (28–68)	176		II and III	4 cycles Doxo + cyclophos (once/3 wks)-4 cycles docetaxel (once/2 wks)	Luminal A: 13.3 Luminal B: 11.9 TN: 34.3 HER2-enriched: 20.7	Luminal A: 67 Luminal B: 45 TN: 35 HER2-enriched: 29
Che [19]	2016	Not specified	50.9 ± 11.0	Pre: 36 Pre and 2 cy: 28		II and III	TA^(4–8)^ or TCAR	19.4	Luminal A: 4 Luminal B: 26 Basal like: 4 HER2-enriched: 2
Bedair [20]	2017	Prospective/single center	Median 53 (32–75)	Pre: 36 2 cy: 22	36		HER2−: Docetaxel^(3)^-FEC^(3)^ 2pt: Taxol-FEC HER2+: FEC^(3)^-Taxol Docetaxel + trastuzumab	38.8	ER+: 24/36 HER2: 13/36
Minarikova [59]	2017	Prospective/single center	52 ± 10 (29–74)	42	42		CA-T^(4+4)^, T-CA^(4+4)^, TA ^(6 or 8)^	16.7	HER2+: 5 TN: 12 ER+ & PR+: 14
Santamaria [27]	2017	Retrospective/single center	54 (27–84)	111			TA^(6)^ (+trastuzumab in HER2+)	18.9	TN: 20 HER2+: 51 ER+/HER2−: 40
Gallivanone [21]	2017	Retrospective/single center	48 ± 12 (28–72)	Baseline: 38 Surgery: 31					Luminal A: 24% Luminal B: 21% HER2-enriched: 13% TN/basal: 42%^f^
Yuan [22]	2018	Prospective/single center	47.3 ± 11.0 (pCR) 43.3 ± 10.0 (non-pCR)^g^	Pre till incl. 6 cy: 142 8 cy: 118		II and III	CA-T^(4+4)^ or T-CA^(4+4)^ TA^(4,6 8)^ Extra to NAC: some cases trastuzumab in HER 2+	28.2	Luminal A: 25 Luminal B: 44 Basal like: 40 HER2-enriched: 33
Partridge [23]	2018	Prospective/multi center	48 ± 10	Pre: 242 Pre & 3 cy: 227 Pre & 12 cy: 210 Pre & post: 186			Pac ± exp agent^(12)^-A^(4)^	33	TN: 77 HER2-enriched: 24 Hormone positive: 141
Kim [53]	2018	Retrospective/not specified	45 (25–67)	46			A/cyclophos A/T A/cyclophos + T A/T + trastuzumab	30.4^h^ (pCR: 10.9)	
Ramirez-Galván [25]	2018	Prospective/single center	48.5 ± 7.8	14	16		Cyclophos + epirubicin^(4)^-Pac^(12)^ Or Clyclosphos + doxorubicin^(4)^-Pac^(12)^ HER2+: trastuzumab Drug toxicity: replace by Carboplatin	25	Hormone+: 7 TN: 5 HER2-enriched: 4
Zhang [24]	2018	Retrospective/single center	52 ± 12.6 (26–73)	61		II and III	Pac + cisplatin HER2: also trastuzumab	39.3	Luminal & HER2+: 30 Luminal & HER2−: 31
Pereira [18]	2019	Prospective/single center	45 (27–65)	62	62		All AC-T based: In HER2: + trastuzumab Or AC-T + carboplatin Or AC-T + (pertuzumab + Trastuzumab and docetaxel)	38.7	TN: 22 HER2-enriched: 10 Luminal B-Ki-67: 23 Luminal B-HER2: 7

*n.r.* not reported, *TN* triple negative, *HER2* human epidermal growth factor receptor 2, *DCIS* ductal carcinoma in situ, *CA-T* anthracycline and cyclophosphamide, followed by taxane, *T-CA* vice versa, *TA* taxane (-based) and anthracycline, *FEC* 5-fluoro-uracil, epirubicin and cyclophosphamide, *T* taxane based, *CAR* carboplatin, *Pac* paclitaxel, *A* anthracycline, *cy* cycles, *base* baseline, *Doxo* doxorubicin, *Cyclophos* cyclophosphamide, *wks* weeks

*Not all studies specified all molecular subtypes

^a^Patients/lesions

^b^11/30 lesions, for two patients no surgery, therefore not included in the 30 lesions

^c^Adriamycin and cyclophosphamide (every 3 weeks), 12 weekly doses of taxanebased OR 4 cycles FEC (once every 3 weeks) followed by 4 cycles taxane based (paclitaxel)

^d^Japanese Breast Cancer Society criteria, grade 3

^e^Tumor regression grade (TRG) 1

^f^The percentage can be too high, see [21]

^g^Overall mean age not reported

^h^Good responders based on Miller and Payne grade 4

**Table 3:** Technical scan parameters

First author	Year	*B*_0_-field (T))/vendor	Reported coil specification	(Acquired/reconstructed) voxel size (mm)	FOV (mm)	TR/TE (ms)	*b* values (s/mm^2^)	Scan moment (s) used for review analysis
Woodhams [64]	2010	1.5 (GE)	Dedicated 8-channel	2.1 × 1.1 × 5	340 × 255	9500/89 (min)	0, 1500	Pre
Tozaki [40]	2010	1.5 (Siemens)	Breast matrix coil	3 × 3 × 3	330	8000/96	500, 1000, 1500, 2000, 3000	Pre, 1 cycle
Fangberget [65]	2011	1.5 (Siemens)	Phased array bi-lateral	1.9 × 1.9 × 4	360 × 195	10,300/126	100, 250, 800	Pre, 4 cycles
Shin [26]	2012	1.5 (Siemens)	4-or 16-channel	3.1 × 1.5 × 3	340	8500/80	0, 100, 500, 800, 1000	Pre, post
Fujimoto [66]	2014	1.5 (Philips)	4 element phased array (SENSE-body)	1.4 × 1.4 × 5	360 × 216	3783/64	0, 800	Post
Hahn [67]	2014	1.5 (GE), 3.0 (Philips)	Surface breast coil	n.r.	n.r.	n.r.	1.5 T: 0, 750	Post
							3.0 T: 0, 1000 and 0, 800	
Bufi [17]	2015	1.5 (GE)	4-channel	FOV 320–340 -> choosing 330: 1.3 × 1.3 × 4	320–340	5150/min (not specified)	0, 1000	Pre
Li [44]	2015	3.0 (Philips)	n.r.	1.3 × 1.3 × 5	192 × 192	(1840–3593)/(43–60)^a^	Different combinations^a^	Pre, 1 cycle
Liu [16]	2015	3.0 (Philips)	Phased array bilateral 8-channel	2.8 × 1.9 × 4	340	7099/51	0, 800	Pre, post
Che [19]	2016	3.0 (GE)	Phased array 8-channel	2.5 × 2 × 5	320 × 320	2400/62.1	0, 10, 20, 30, 50, 70, 100, 150, 200, 400, 800, 1000	Pre, 2 cycles
Bedair [20]	2017	3.0 (GE)	Dedicated 8-channel phased array coil	2.7 × 2.7 × 4	350 × 350	5000/77^b^	0, 30, 60, 90, 120, 300, 600, 900	Pre, 2 cycles
Minarikova [59]	2017	3.0 (Siemens)	Bilateral breast 4	1.4 × 1.4 × 5	n.r.	5800/68	0 and 850	Pre, 2, 3 & 4, 5 cycles
			^1^H-channels					
Santamaria [27]	2017	1.5 (GE)	4-channel breast surface coil (GE)	2.4 × 2.4 × 4	Aera: 360 × 270	Aera: 6500/66	Aera: 50, 700	Pre, post
		1.5 (Siemens)	16-channel breast surface coil (Siemens)		Signa: 320 × 320	Signa: 8000/65	Signa: 0, 700	
Gallivanone [21]	2017	1.5 (Philips)	7-channel	1.4 × 1.4 × 3	310 × 310	10,000/66	0, 900	Pre
Yuan [22]	2018	3.0 (GE)	Phased array 8-channel	2.3 × 1.6 × 5	300 × 250	2400/62	0, 300, 600, 1000	Pre, 1 cycle (but multiple in full-text)
Ramirez-Galván [25]	2018	1.5 (GE)	Bilateral 8-channel	2.5 × 2.5 × 3	320	4825 (3000–6000) /87.9	0, 700	Pre, 1, 2, 3 cycles, post
Partridge [23]	2018	1.5, Philips, 3.0 Siemens, GE	Dedicated RF-coil	1.88 × 1.88 × 4^c^	300–360	> 4000/min	0, 100, 600, 800	Pre, 3 weeks, 12 weeks, post
Kim [53]	2018	3.0 (Siemens)	Dedicated surface breast coil	1.77 × 0.89 × 4	340 × 170	5600/55	0, 25, 50, 75, 100, 150, 200, 300, 500, 800	Pre, 2 cycles
Zhang [24]	2018	3.0 (Philips)	Dedicated 4-channel array	1.25 × 1.25 × 3	230 × 240	2681/82	0, 800	Pre, 2 cycles
Pereira [18]	2019	1.5 (GE, Philips)	Dedicated 8-channel	n.r.	n.r.	n.r.	0, 750	Pre, 1 cycle, post

*TE* echo time, *TR* repetition time, *FOV* field-of-view

^a^Different TR/TE and *b*-value combinations (0, 500 s/mm^2^ or 0, 600 s/mm^2^ or 50, 600 s/mm^2^)

^b^In full-text TE = 5.0 ms → within review interpreted as seconds

^c^Defined as range, here chosen for max FOV and max acquired matrix and min slice thickness

**Table 4:** Studies classified by pCR-definition

pCR-definition/first author	pCR rate (95% CI)	N^a^	Total^b^
*No invasive. No DCIS*			
Santamaria [27]	0.19 (0.12–0.26)	21	111
Minarikova (baseline) [59]	0.17 (0.05–0.28)	7	42
Minarikova (after 5 cycles) [59]	0.15 (0.03–0.27)	5	33
Woodhams [64]^c^	0.13 (0.05–0.21)	9	70
*No invasive. DCIS may be present*			
Che [19]	0.19 (0.07–0.32)	7	36
Bedair [20]	0.39 (0.23–0.55)	14	36
Fangberget [65]	0.37 (0.19–0.54)	11	30
Shin [26]	0.37 (0.22–0.51)	15	41
Hahn [67]	0.24 (0.15–0.34)	19	78
Yuan [22]	0.28 (0.21–0.36)	40	142
Partridge (pre) [23]	0.31 (0.25–0.37)	71	227
Partridge (mid) [23]	0.33 (0.27–0.40)	70	210
Partridge (post) [23]	0.34 (0.27–0.41)	63	186
Gallivanone [21]^d^	0.42 (0.25–0.59)	13	31
Fujimoto [66]	0.14 (0.05–0.23)	8	56
Woodhams [64]^e^	0.13 (0.23–0.33)	16	70
Pereira [18]	0.39 (0.27–0.51)	24	62
*No invasive (without specification)*			
Bufi [17]	0.17 (0.12–0.22)	39	225
Ramirez-Galván [25]^f^	0.25 (0.04–0.46)	4	16
Li [44]	0.33 (0.19–0.48)	14	42
*Near pCR*			
Liu [16]^g^	0.18 (0.12–0.24)	32	176
Kim [53]^g^	0.30 (0.17–0.44)	14 (5: real pCR)	46
*No definition for pCR*			
Tozaki [40]	0.14 (0–0.40)	1	7

*pCR* pathologic complete response, *DCIS* ductal carcinoma in situ, *CI* confidence interval

^a^Lesions/patients with a pCR

^b^Total lesions/patients

^c^Data extracted from supplementary material

^d^Calculated from 42% pCR from full-text

^e^Broader pCR-definition including DCIS, in addition to original pCR-definition in full-text. Data extracted by supplementary materials

^f^Miller and Payne grade 5

^g^Miller and Payne 4 included, in Kim et al. [53] was labeled as good responders

### MRI characteristics and DWI measures to predict and evaluate NST response

Regarding MRI-scanners, coils, and acquisition parameters of the DWI sequence, large heterogeneity was observed (Table 3). For example, in ten studies, DWI was performed at 1.5 T, eight studies used a 3.0 T scanner, and two studies used MRI-scanners with both field strengths. Although most studies used single-shot echo-planar imaging (SS-EPI), a wide variety was observed within and between studies regarding echo times (TE), the use of low *b*-values (< 150 s/mm^2^), methods to calculate ADC-values, and region of interest (ROI)-definitions (Table 5). Details/study characteristics (Tables 2, 3) are reviewed in “Discussion” section.

**Table 5:** Main region-of-interest specifications

First author	2D/3D	Nr. (one/multiple)^1^	Predefined absolute size?	Excluding areas^2^	Only highest signal (*b*-image)/lowest on ADC map/solid part tumor^3^	ROI no residual disease post-NST visible
Santamaria [27]^4^	2D: circular	Three (diff. sections)	Y: (≤ 15 mm^2^)	Y	Not specified	Pretreatment location
Tozaki [40]	2D: circular	One	Y 19.6 mm^2^ (*r* = 2.5 mm)	n.r.	Y	n.r.
Bufi [17]						
– Before	2D	One	No	Y	No	N/A
– Post^5^	–	–	–	–	–	
Che [19]	2D	One	No: based on max transverse diameter	Y	Different description	n.r.
Fangberget [65]	Not spec.	One (solid part)	No	Y	Y	n.r.
Minarikova [59]^6^	3D	One	No: region growing (upper & lower bounds)	Not specified (in 3D)	No	n.r.
Woodhams [64]^7^	2D	Pre: two to seven	No	Not specified	No	n.r.
		Post: one to seven				
Shin [26]	3D	One	No	Y	No	No residual enhancement: images compared pre and post-NAC, incl. cardiac level and the surrounding
Hahn [67]	2D	Three (slices)	No (based on the largest cross-sectional planes → three slides)	Y (especially fat and normal parenchyma, further not specified)	No	n.r.
Yuan [22]	3D	One	No	Y	No	n.r.
Partridge [23]	3D	One composite	No	Y	No	Region at previous scan with visible tumor
Gallivanone [21]	3D	One	No (semi-automatic method: see Gallivanone et al.)	Y	Not only (see details Gallivanone et al.)	n.r.
Li [44]	3D	One	No (copied from the DCE-ROI tumor^8^)	n.r.	Different description	n.r.
Fujimoto [66]	2D	One	No (based on largest diameter)	Y	Different description	Pre-treatment ROI
Liu [16]	2D (pseudo- 3D)	Three	No (based on largest cross-sectional area’s)	n.r.	Different description	Pre-treatment ROI
Bedair [20]	2D	One	No	Y	No: largest tumor dimension on *b* = 900	n.r.
Ramirez-Galván [25]	2D	Three	No (three ellipses randomly placed)	Y	No	?
Pereira [18]	2D	One	n.r.	Y	Y	n.r.
Zhang [24]	2D	Three types:	n.r.	Y	Different description	n.r.
		(a) Freehand				
		(b) Single-round				
		(c) Three-round				
Kim [53]	2D (manual) → 3D (automatic)	Three (sagittal, coronal, axial)	No	Y	Different (on *b* = 0, based on DCE and T2)	n.r.

*ADC* apparent diffusion coefficient, *n.r.* not reported, *r* radius, *ROI* region of interest

^1^Short description

^2^Not specified for which areas (but referring to areas such as inner margins, necrotic, fibrotic areas etc.)

^3^No: the ROI was not limited to solid or other tumor part on the slice or high signal on *b*-image, respectively, low signal on ADC

^4^Multiple ROI methods, but this refers to mean value method used for data analysis, the reported area could be for all three together or each area

^5^Situation depended: delineation on *b* = 1000 s/mm^2^, in case of tumor fragmentation ROI including not hyperintense “interspersed” area and the whole lesion, otherwise in case of no clear region, ROI of 100 pixels within the previous observed area

^6^2D and 3D ROI’s: 3D-ROI’s in majority used for comparisons-not applicable: referring to a different image property

^7^The ADC of the multiple ROI’s were averaged and mean ROI-size was pre-NST 37 ± 17 mm and post-NST 20 ± 15 mm

^8^At least 80% percent signal intensity increase after contrast injection, see further Li et al. [44] Pseudo 3D: several slices, but not whole lesion in 3D

**Fig. 2:**
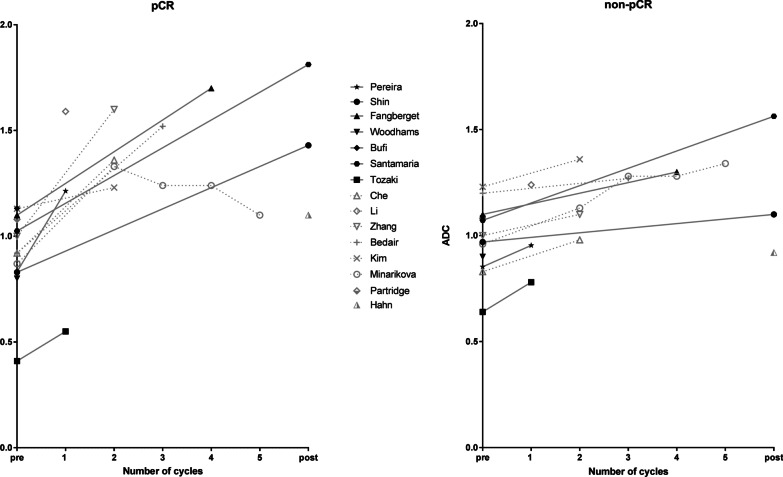
Mean/median ADC-values (× 10^−3^ mm^2^/s) in different studies pre, during and post-NST between the pCR group (left figure) and the non-pCR group (right figure). Different time points are connected by solid (: studies acquired at 1.5 T), dashed (: studies acquired at 3.0 T) lines. Hahn et al. and Partridge et al. used both 1.5 T and 3.0 T scanners represented with non-connected points. The legend shows different studies that are included in the graphs. Note: the period of a cycle of neoadjuvant therapy (number of weeks) can differ and within and between studies as well as the total number of cycles. Subsequently, the solid, and dashed arrow lines should not be used for interpolation of ADC-values between two measuring time points. For Woodhams et al. [64] only the pCR-definition from the full-text was used, ADC rounded at one decimal. For Kim et al. [53] Miller and Payne grade 4 as good responders included

**Fig. 3:**
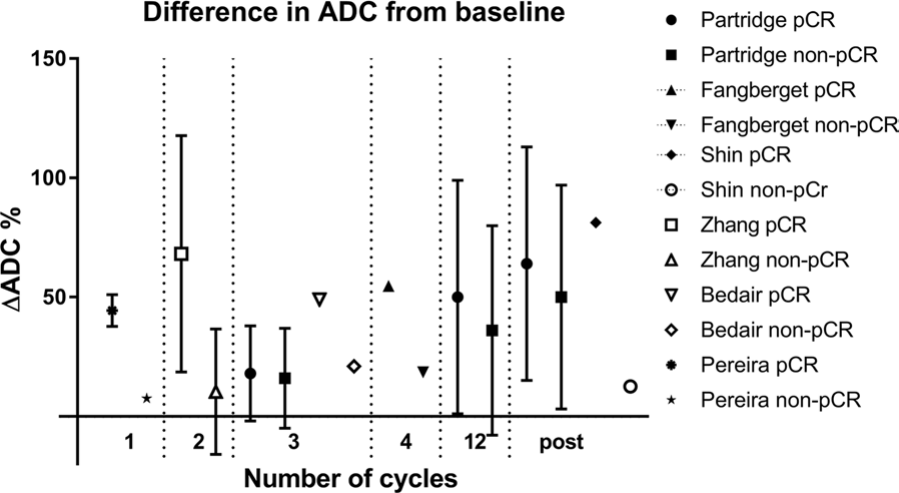
Studies reporting the percentage difference in ADC for pCR and non-pCR from baseline for the general study population at different time points. *Note*: The period of a cycle of neoadjuvant therapy (number of weeks) can differ within and between studies as well as the total number of cycles

Furthermore, the DWI measures varied in the studies (e.g., absolute, relative (: (percentage) change, ratios) or histogram related values). Figures 2 and 3 illustrate the ADC values and the percentage change in ADC over time for pCR and non-pCR, respectively. In Fig. 2, studies using scanners with the main magnetic field strength *B*_0_, 1.5 T or 3 T, were also visually separated (Fig. 2).

#### Baseline DWI–MRI

**Fig. 4:**
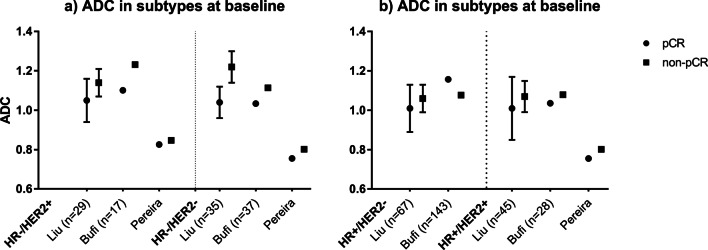
ADC-values (× 10^−3^ mm^2^/s) at baseline per molecular subtype for two of the included studies, with two subtypes (HR-) in **a** and two subtypes (HR+) in **b**. Bufi et al. [17] distinguished triple negative, HER2-enriched, luminal, hybrid (: luminal and HER2+, HR+/HER2+) tumors. Liu et al. [16] distinguished luminal A (ER+ and/or PR+ incl. Ki67 < 14% or HER2−), luminal B (ER+ and/or PR+ incl. Ki67 ≥ 14% or HER2+), HER2-enriched and triple negative tumors. In this graph, the types from Liu et al. [16] of luminal A are appointed as HR+/HER2− and luminal B as HR+/HER2−. From Bufi et al. [17] the luminal group is appointed as HR+/HER2−. From Pereira et al. [18] three subtypes were reported

**Fig. 5:**
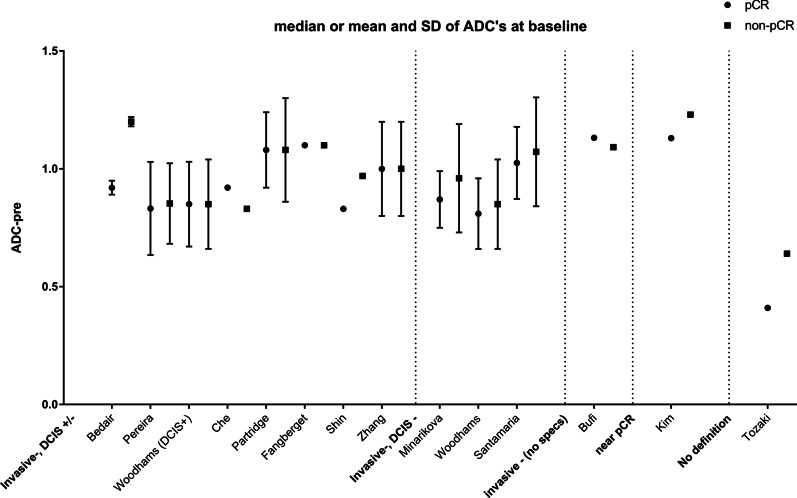
Mean/median ADC (× 10^−3^ mm^2^/s) at baseline for pCR and non-pCR (and if known, the standard deviation), using different sub-classifications for pCR. For Woodhams et al. [64] mean and standard deviation extracted from data supplementary material, rounded by two decimals for both pCR-definitions: with and without DCIS

Sixteen publications investigated DWI–MRI at baseline for predicting pCR. Most studies tried to identify an ADC-threshold. The reported overall (mean or median) ADC-values varied between studies for patients that obtained pCR (0.41 × 10^−3^–1.16 × 10^−3^ mm^2^/s) and those that did not (0.64 × 10^−3^–1.23 × 10^−3^ mm^2^/s). Reported thresholds were highly variable. Figure 4 shows the results of three studies that distinguished pCR/non-pCR based on molecular subtype [16–18]. In general intervals of ADC-values for pCR and non-pCR cases were overlapping between studies (Fig. 5). An observed trend within studies, where residual DCIS is explicitly not allowed in the pCR-definition, is that some tumors with a relative low ADC tend to have a higher chance to show pCR on NST (Fig. 5, category: “Invasive-, DCIS-”).

Some studies reported non-mono-exponential/non-Gaussian models, (e.g., intravoxel incoherent motion (IVIM)). A mean true diffusion coefficient (*D*) of 0.92 × 10^−3^ mm^2^/s (pCR) versus 0.83 × 10^−3^ mm^2^/s (non-pCR) was reported (*p* = 0.323) [19]. Another non-Gaussian approach, stretched exponential modeling (SEM), quantifying the intravoxel heterogeneity (i.e., the intravoxel heterogeneity index (*α*)) and the distributed diffusion coefficient (DDC) in a multi-exponential decay, resulted in cutoff values for *α* = 0.838 (AUC = 0.644) and DDC = 1.141 × 10^−3^ mm^2^/s (AUC = 0.756) [20]. Furthermore, one study identified skewness (*p* < 0.05) and entropy (*p* = 0.05) (both histogram based features) as predictor for pCR [21]. More details are presented in Table 6.

**Table 6:** DWI parameters pre-NST

First author	ADC-value (× 10^−3^ mm^2^/s) pCR versus non-pCR^a^	Reported/chosen ADC threshold for pCR (× 10^−3^ mm^2^/s)	ROC AUC (95% CI)	Sensitivity (%)	Specificity (%)	PPV (%)	NPV (%)
Minarikova [59]	0.87 ± 0.12 versus 0.96 ± 0.23 *p* = 0.287		0.633				
Shin [26]	0.83 (0.77, 0.87) versus 0.97 (0.82, 1.10) *p* = 0.014	0.92	0.75 (0.58, 0.88)	82	65		
Fangberget [65]^b^	1.1 versus 1.1 *p* = 0.693		0.80				
Woodhams [64]^c^	pCR as in full-text (excl. DCIS)		0.55				
	0.81 ± 0.15 versus 0.85 ± 0.19 *p* = 0.64		0.52				
	pCR incl. DCIS: 0.85 ± 0.18 versus 0.85 ± 0.19 *p* = 0.82						
Bufi [17]^d^	Overall: 1.132 versus 1.092 *p* = 0.23	Overall: 0.975	Overall: 0.587				
	Luminal: 1.157 versus 1.077 *p* = 0.59	Luminal: 0.832	Luminal: 0.588				
	Hybrid: 1.036 versus 1.079 *p* = 0.53	Hybrid: 0.959	Hybrid: 0.567				
	TN: 1.034 versus 1.114 *p* = 0.06	TN: 0.995	TN: 0.766				
	HER2+: 1.101 versus 1.232 *p* = 0.05	HER2+: 0.971	HER2+: 0.813				
Pereira [18]	Overall: 0.832 ± 0.198 versus 0.853 ± 0.171 *p* = 0.882						
	Luminal B: 0.755 (0.596–1.035) versus 0.802 (0.483–1.090) *p* = 0.359						
	TN: 0.857 (0.448–1.330) versus 1.02 (0.739–1.390) *p* = 0.070						
	HER2: 0.826 (0.651–1.140) versus 0.847 (0.772–0.949) *p* = 0.522						
Santamaria [27]	1.025 ± 0.153 versus 1.072 ± 0.231 *p* = 0.549						
Li [44]		1.22	0.72	93	52	50	
Tozaki [40]^e^	0.41 versus 0.64 (range 0.46–0.83)	0.45	–	100	100		
Che [19]^f^ (IVIM- > D)	0.92 (0.77, 0.95) versus 0.83 (0.75, 0.92) *p* = 0.323	0.874	0.600 (0.424–0.759)	69.2 (38.6–90.9)	65.2 (42.7–83.6)	52.9 (28.5–76.1)	78.9 (53.9–93.0)
Kim [53]^g^	1.13 (1.01–1.25) versus 1.23 (1.12–1.41) → ADC						
	1.10 (1.01–1.22) versus 1.22 (1.10–1.49) → D						
Yuan [22]			Luminal A: 0.556				
			Luminal B: 0.538				
			Basal-like: 0.534				
			HER2-Enr.: 0.601				
Partridge [23]	1.08 ± 0.16 versus 1.08 ± 0.22						
Liu [16]	Luminal A: 1.01 ± 0.12 versus 1.06 ± 0.07 *p* = 0.293						
	Luminal B: 1.01 ± 0.16 versus 1.07 ± 0.08 *p* = 0.070						
	HER2-enriched: 1.05 ± 0.11 versus 1.14 ± 0.07 *p* = 0.098						
	Triple-negative: 1.04 ± 0.08 versus 1.22 ± 0.08 *p* < 0.001						
Bedair [20]	0.92 ± 0.03 versus 1.20 ± 0.02 *p* < 0.01 → ADC	1.012	0.749	81	67		
	0.93 ± 0.04 versus 1.25 ± 0.03 *p* < 0.01 → DDC	1.141	0.756	81	72		
	0.85 ± 0.05 versus 1.02 ± 0.05 *p* = 0.02 → D	0.838	0.644	60	47		
	Other model based measures: 0.81 ± 0.02 versus 0.84 ± 0.02 *p* = 0.07 → *α* (a.u.)	0.967	0.641	71	53		
Zhang [24]	1 ± 0.2 versus 1 ± 0.2 *p* = 0.645						

*ADC* apparent diffusion coefficient, *CI* confidence interval, *D* true diffusivity, *DCIS* ductal carcinoma in situ, *DDC* distributed diffusion coefficient, *HER2* human epidermal growth factor receptor 2, *f* perfusion fraction, *ROC AUC* area under the receiver operating characteristic curve, *NPV* negative predictive value, *pCR* pathologic complete response, *PPV* positive predictive value, *TN* triple negative

^a^Mean ADC-value ± SD with the exception of Che et al. [19]: median ADC and the interquartile range

^b^31 MRI at pre NAC and after 4 cycles 27 MRI’s

^c^Mean and SD calculated by data extraction within the supplementary material, rounded by two decimals, *p* value calculated with independent samples Mann–Whitney *U* test, and AUC-ROC in SPSS

^d^Hybrid tumors: luminal tumors with HER2+; TN: triple negative; data from the HER2+ group represents the HER2-enriched tumors in this case

^e^Threshold can be chosen based on ADC-value of the pCR case, resulting in 100% sensitivity and specificity

^f^D is the true diffusion coefficient in IVIM

^g^Miller and Payne grade 4 included as good responders

#### DWI–MRI during NST

Nine studies reported on absolute ADC-values during NST to predict pCR. The scan-moments varied widely between the studies (after 1–5 NST-cycles). Reported ADC-values were heterogeneous. Overall, increasing ADC-values during NST seem to reflect response of the tumor. However, there is no clear threshold to distinguish partial and non-responders from complete responders. The optimal scan-moment evaluating therapy during NST seems to be subtype and NST-regimen dependent.

In one study [22], three types of NST (start) regimens were compared to predict pCR for different molecular subtypes. Looking at the highest AUC per subtype over all NST variants, the optimal scan-moment for pCR prediction in Luminal A and B after starting with taxanes or anthracyclines is suggested after 3 weeks of therapy. When using change in ADC, an AUC = 0.865 for Luminal B (starting with taxanes) and AUC = 0.845 for luminal A (when starting with anthracyclines) are reported. The optimal scan-moment for basal-like and HER2-enriched tumors starting with anthracyclines and taxanes is suggested after 3 weeks, with AUC = 0.879 and AUC = 0.783, respectively, using change in ADC. For other NST-regimen and molecular subtype combinations, 6 weeks is reported as optimal scan-moment. The optimum can thus differ, depending on a specific NST-type and cancer subtype; see for all details [22].

A difficulty is that reported series are in general small. Subdividing those in different subtypes and NST regimen leads to very small study populations. Partridge et al. [23] reported that all subtypes were underpowered, except HR+/HER2−. For this subtype, the predictive value of DWI ($\Delta$ADC (%)) after 3 weeks of taxane (paclitaxel) treatment achieved an AUC of 0.61, whereas Yuan et al. [22] reported an AUC = 0.678 for the (absolute) $\Delta$ADC in Luminal A cancers, neglecting Ki-67 in this comparison. Furthermore, one study investigated three ROI-types in luminal cancer and defined the optimal ROIs according to the specific shrinkage pattern, achieving an AUC = 0.877 for $\Delta$ADC% after two cycles [24]. In addition, ADC-ratios, related to baseline and a time point (number of cycles), were analyzed. Here, increased AUCs were observed as the evaluation moment progressed toward post-NST [25].

Studying IVIM, Che et al. [19] found after two cycles a mean true diffusion coefficient (*D*) of 1.36 × 10^−3^ mm^2^/s (pCR) versus 0.98 × 10^−3^ mm^2^/s (non-pCR) over all subtypes (*p* = 0.001). For distinguishing pCR/non-pCR, they reported a cutoff value of 0.971 × 10^−3^ mm^2^/s, yielding a 100% sensitivity at 63% specificity (AUC = 0.851). Another IVIM-parameter, the change in perfusion fraction ($\Delta f)$ showed an AUC of 0.906 using a cutoff of 11.3% [19]. More details are displayed in Tables 7 and 8.

**Table 7:** DWI parameters during NST

First author	Cycle (s)	ADC-value (× 10^−3^ mm^2^/s) pCR versus non-pCR^a^	Reported/chosen ADC threshold for pCR (× 10^−3^mm^2^/s)	ROC AUC	Sensitivity (%)	Specificity (%)	PPV (%)	NPV (%)
Tozaki [40]	1	0.55 versus 0.78 (range 0.45–0.95)						
Li [44]	1	1.59 versus 1.24 *p* = 0.0019	1.4	0.82	83	67	59	
Pereira [18]	1	1.214 ± 0.0599 versus 0.954 ± 0.0267						
Che [19] (IVIM)	2	1.36 ± 0.30 versus 0.98 ± 0.23 *p* = 0.001	0.971	0.851	100 (66.4–100)	63.2 (38.4–83.7)	56.3 (30.6–79.3)	100 (69.9–99.2)
Zhang [24]	2	1.6 ± 0.4 versus 1.1 ± 0.3 *p* < 0.001		0.864				
Kim [53]^b^	2	ADC: 1.23 (1.10–1.38) versus 1.36 (1.32–1.57)	1.29	0.70	0.79	0.62		
		D: 1.15 (1.10–1.34) versus 1.37 (1.25–1.60)	1.35	0.71	0.71	0.77		
Minarikova [59]^c^	2^c^	1.33 ± 0.28 versus 1.13 ± 0.26		0.697^d^				
	3 & 4^c^	1.24 ± 0.15 versus 1.28 ± 0.30		0.500				
Bedair [20]	3	1.52 ± 0.32 versus 1.27 ± 0.18 → ADC						
		Other model-based metrics:						
		1.51 ± 0.15 versus 1.40 ± 0.12 → DDC						
		1.30 ± 0.14 versus 1.28 ± 0.15 → D						
		0.91 ± 0.07 versus 0.86 ± 0.11 → α (a.u.)						
		8.48 ± 1.54 versus 10.53 ± 2.51 → f (%)						
Fangberget [65]^e^	4	1.7 (range: 1.0–2.1) versus 1.2 (range: 0.9–1.7) or 1.3 *p* = 0.022^f^	1.42		88	80		
Minarikova [59]^c^	5	1.10 ± 0.24 versus 1.34 ± 0.33		0.743				

*ADC* apparent diffusion coefficient, *D* true diffusivity, *DDC* distributed diffusion coefficient, *f* perfusion fraction, *NPV* negative predictive value, *PPV* positive predictive value, *pCR* pathologic complete response, *ROC AUC* area under the receiver operating characteristic curve, *TN* triple negative, *α* intravoxel heterogeneity index

^a^Mean ADC-value ± SD with the exception of Li et al. [44]: median ADC

^b^Miller and Payne grade 4 included as good responders

^c^After 2 cycles: 14 lesions; after 3–4 cycles: 19 lesions and 5 cycles: 34 lesions scanned

^d^About the data (ADC) and ROC-analysis: “smaller values were considered positive for pCR prediction in BS, after three to four cycles and after five to eight cycle; however, higher values were considered positive for pCR prediction in data measured after two cycles” [59]

^e^31 MRI at pre NAC and after 4 cycles 27 MRI’s

^f^1.2 × 10^−3^ mm^2^/s and 1.3 × 10^−3^ mm^2^/s for non-pCR mentioned in [65]

**Table 8:** Change in ADC between baseline and during NST; (i) percentage change, (ii) absolute change, (iii) ADC ratios baseline

First author	After *N* cycles	pCR versus non-pCR (mean ± SD%)	ADC percentage change cutoff (%)	AUC	Sens (%)	Spec (%)	PPV (%)	NPV (%)
(i) *ΔADC %*								
Li [44]	1		6.5	0.63	50	78	55	
Pereira [18]	1	Overall: 44.36 ± 6.7 versus 7.54 ± 2.3 *p* = < 0.001						
		TN: 53 versus 7 *p* = 0.002						
		Luminal B: 42 versus 16 *p* = 0.009						
		HER2-ov.exp: 43 versus 7 *p* = 0.055						
Zhang [24]	2	68.2 ± 49.6 versus 10.4 ± 26.3		0.877				
Partridge [23]	3 (= 3 weeks)	Overall: 18 ± 20 versus 16 ± 21; *p* = 0.48		0.53				
		HR−/HER2−: 14 ± 15 versus 15 ± 18; *p* = 0.94		0.51				
		HR+/HER2−: 22 ± 18 versus 15 ± 22; *p* = 0.18		0.61				
		HR−/HER2+: 25 ± 26 versus 32 ± 28; *p* = 0.52		0.61				
		HR+/HER2+: 14 ± 23 versus 18 ± 23; *p* = 0.43		0.58				
Bedair [20]	3	49 versus 21 *p* = 0.03 → ADC						
		Other model based metrics:						
		45 versus 32 *p* = 0.04 → DDC						
		36 versus 23 *p* = 0.14 → D						
		− 29 versus 5 *p* = 0.05 → f						
		7 versus 5 *p* = 0.68 → α						
Fangberget [65]*	4	54.7 versus 18.5 *p* = 0.111						
Partridge [23]	12 (= 12 weeks)	Overall: 50 ± 49 versus 36 ± 44; *p* = 0.017		0.60				
		HR−/HER2−: 33 ± 36 versus 26 ± 40; *p* = 0.33		0.57				
		HR+/HER2−: 75 ± 43 versus 35 ± 40; *p* < 0.001		0.76				
		HR−/HER2+: 63 ± 65 versus 35 ± 57; *p* = 0.40		0.67				
		HR+/HER2+: 40 ± 43 versus 56 ± 56; *p* = 0.53		0.56				

First author	After *N* cycles	pCR versus non-pCR (× 10^3^ mm^2^/s)^1^	ADC change cutoff (× 10^3^ mm^2^/s)	AUC	Sens (%)	Spec (%)	PPV (%)	NPV (%)
(ii) *ΔADC*								
Yuan [22]**	1		Luminal A^2^: 0.5589	0.845	87.3	73.4		
			Luminal B^3^: 0.5746	0.865	89.4	83.4		
			Basal-like^4^: 0.5854	0.879	89.9	82.6		
			HER2 enr.^4^: n.r.	0.783	n.r.	n.r.		
Che [19]^5^	2	− 0.45 (− 0.67, − 0.29) versus 0.07 (− 0.16, − 0.01) *p* < 0.001	− 0.163	0.924	100	73.7	64.3	100

First author	Time points	pCR versus non-pCR^6^	ADC ratio cutoff (A.U.)	AUC	Sens (%)	Spec (%)	PPV (%)	NPV (%)
(iii) *ADC-ratio of two time points*								
Ramirez-Galván [25]	1 cycle/pre	1.08 ± 0.4 versus 1.12 ± 0.09	≤ 1.09	0.641	85.9	58.6		
	2 cycles/pre	1.30 ± 0.28 versus 1.10 ± 0.10	> 1.14	0.807	79.2	79.7		
	3 cycles/pre	1.35 ± 0.28 versus 1.10 ± 0.15	> 1.08	0.826	100	66.7		
	Post/pre	1.49 ± 0.20 versus 1.13 ± 0.01	> 1.25	0.938	100	83.8		

*ADC* apparent diffusion coefficient, *AUC* area under the curve, *HER2-enr.* human epidermal growth factor receptor 2 enriched, *HR* hormone receptor, *pCR* pathologic complete response, *Sens* sensitivity, *Spec* specificity, *PPV* positive predictive value, *NPV* negative predictive value, *α* intravoxel heterogeneity index, *Δ* representing change

*31 MRI at pre NAC and after 4 cycles 27 MRI’s

**Data in full-text was reported based on different NST (started with taxanes or anthracyclines, or taxanes and anthracyclines) and the molecular subtypes

^1^There has been chosen to use the exact numbers (positive and negative) in order to avoid misinterpretation, when definitions are not mentioned in the full-text

Median and interquartile range in change in ADC for Che et al. [19]

^2^Compared after 1 cycle with anthracyclines

^3^Compared after 1 cycle of taxanes

^4^Compared after 1 cycle of anthracyclines and taxanes

^5^(Parameter-baseline)–(parameter after two cycles), change in true diffusion (D)

^6^Ratio: ADC time point after baseline/ADC baseline

#### DWI–MRI after NST

Four papers evaluated absolute post-NST ADC-values (Table 9). In one study [26], an ADC-threshold of 1.19 × 10^−3^ mm^2^/s to distinguish pCR/non-pCR yielded an AUC of 0.80. Another study [16] used higher thresholds that also differed for the molecular subtypes (range: 1.33 × 10^−3^ mm^2^/s (luminal B) to 1.43 × 10^−3^ mm^2^/s (triple negative)).

Using the change in ADC between baseline and post-NST, one study suggested a threshold of 40.7% of increase to identify patients with a pCR, with 100% sensitivity, 91% specificity, and an AUC of 0.96 [26].

Another measure, the ADC-ratio (= mean post-ADC/mean pre-ADC), used in Santamaria et al. [27] was significant (*p* = 0.009) for distinction pCR/non-pCR (AUC = 0.73) (Table 10).

**Table 9:** DWI parameters after NST

First author	ADC-value (× 10^−3^ mm^2^/s) pCR versus non-pCR^a^	Reported/chosen ADC threshold for pCR (× 10^−3^ mm^2^/s)	ROC AUC (95% CI)	Sens (%)	Spec (%)	PPV (%)	NPV (%)
Shin [26]	1.43 (1.24, 1.69) versus 1.10 (0.93, 1.23) *p* = 0.003	1.19	0.80 (0.62, 0.94)	100	70		
Santamaria [27]	1.812 ± 0.294 versus 1.563 ± 0.471 *p* = 0.011	n.r.					
Hahn [67]		1.10 ± 0.54 versus 0.92 ± 0.33 *p* = 0.130		65.0	91.4	72.2	88.3
Liu (broad pCR-definition) [16]	Luminal A: 1.39 ± 0.07 versus 1.15 ± 0.09 *p* < 0.001	Luminal A: 1.35	0.864	75.0	96.6	75.0	96.6
	Luminal B: 1.41 ± 0.12 versus 1.17 ± 0.07 *p* < 0.001	Luminal B: 1.33	0.857	71.4	97.4	71.4	94.9
	HER2 enriched: 1.39 ± 0.07 versus 1.24 ± 0.08 *p* < 0.001	HER2-enriched: 1.38	0.792	62.5	95.2	83.3	87.0
	TN: 1.44 ± 0.09 versus 1.33 ± 0.06 *p* < 0.001	TN: 1.43	0.751	66.7	82.6	66.7	82.6
Woodhams [64]	Visual assessment^b^	Visual	Visual	89	97	80	98

*ADC* apparent diffusion coefficient, *HER2* human epidermal growth factor receptor 2, *CI* confidence interval, *pCR* pathological complete response, *Sens* sensitivity, *Spec* specificity, *PPV* positive predictive value, *NPV* negative predictive value, *TN* triple negative

^a^Mean ADC-value ± SD with the exception of Shin et al. [26]: median ADC and the interquartile range

^b^Residual disease was defined positive in full-text, in the table above pCR is defined positive. PPV and NPV were calculated from extracted data of the full-text. Performance regarding visual assessment. Post-NST ADC-values could not be extracted for the whole study population

More details about change in ADC are displayed in Fig. 3 (three studies at different time points) and Table 10.

**Table 10:** Percentage change in ADC after NST

First author	After *N* cycles	pCR versus non-pCR (mean ± SD%)	ADC percentage change cutoff (%)	ADC ratio pCR versus non pCR^a^	AUC
*ΔADC %*					
Santamaria [27]	Post (~ 6 cycles)			1.788 ± 0.299 versus 1.487 ± 0.473 *p* = 0.009	0.73
Shin [26]	Post	81.3 versus 12.6 *p* < 0.001	40.7^b^		0.96
Partridge [23]	Post	Overall: 64 ± 49 versus 50 ± 47; *p* = 0.013			0.61
		HR−/HER2−: 68 ± 32 versus 39 ± 39; *p* < 0.001			0.75
		HR+/HER2−: 82 ± 41 versus 54 ± 50; *p* = 0.01			0.71
		HR−/HER2+: 63 ± 79 versus 28 ± 46; *p* = 0.56			0.62
		HR+/HER2+: 43 ± 37 versus 61 ± 47; *p* = 0.64			0.55

*ADC* apparent diffusion coefficient, *AUC* area under the curve, *HER2* human epidermal growth factor receptor 2, *HR* hormone receptor, *pCR* pathological complete response, Δ representing change

^a^$$\frac{{\text{ADC}}_{{\text{mean}}\, {\text{post}}}}{{\text{ADC}}_{{\text{mean}} \,{\text{pre}}}}$$

^b^corresponding to a sensitivity of 100% and specificity of 91%

Finally, also the ROI-methodology differed between studies for cases with and without apparent residual disease (ROI-specifications: Table 5).

## Discussion

This review describes 20 studies reporting on DWI–MRI prior to/during/after NST to identify pCR of the breast. A major finding is that the studies were very heterogeneous regarding clinical, technical, and epidemiological aspects. These differences make pooling of results for meta-analysis difficult. Previous meta-analyses [12, 13] should therefore be interpreted with caution. Currently, it is impossible to define the role of DWI in identifying pCR after NST. The observed heterogeneity in type of cancers, applied treatments, and used quantification methods precludes straightforward implementation of DWI protocols for NST-monitoring in other hospitals.

Some of these limitations were also recognized for the value of DWI for lesion classification. The European Society of Breast Imaging (EUSOBI) International DWI working group recently published a consensus and mission statement to alleviate this issue for lesion classification only [14]. Further standardization to implement DWI for treatment monitoring seems based on the findings of current systematic review essential.

The Quantitative Imaging Biomarkers Alliance (QIBA) of the RSNA published in 2018 for some organs standards related to implementation of quantitative DWI biomarkers (like reproducibility, repeatability, and regarding measurement errors vs. real changes) [28]. In the revised standard, currently under development, also technical breast imaging aspects are included [29]. These aspects may alleviate some differences in acquisition and evaluation parameters that currently make multicenter implementation challenging. From a technical perspective, even more parameters than discussed in this review may influence measurements [30–33]. Different hardware components and MRI-protocols might also initiate effect on the precision and accuracy of the DWI metrics obtained for pCR prediction/evaluation or even DWI in general [34–36]. Furthermore, interpretation factors (e.g., reading system, reader experience) may affect results. Some quality issues were already addressed in a test–retest study of Newitt et al. [37]. Strikingly, the biological variability of cancers and the differences in treatment protocols are not at all addressed by the available guidelines.

Below, we discuss some of the most eye-catching differences between studies with respect to treatment monitoring that need to be addressed shortly. We acknowledge that this list is certainly not complete.

**Fig. 6:**
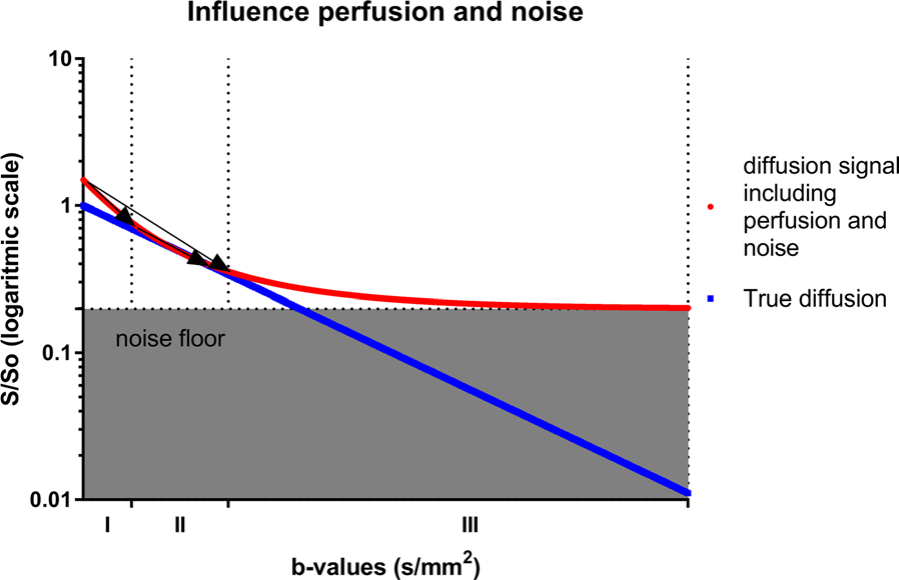
Schematic overview, with the semi logarithmic plots (and S0, the signal at *b* = 0 without perfusion component) of the signal attenuation of pure diffusion (blue curve) and signal attenuation by (micro)perfusion, diffusion and including contribution of noise by the rician noise floor (red curve). Within the red curve, the first small arrow represents the mono-exponential slope (ADC1) within segment I, the second small arrow includes the mono-exponential slope in segment II (with ADC2). The large arrow represents the mono-exponential approach/slope (ADC3) using two *b*-values, one in segment I and one in segment II. Three segments of diffusion sensitive gradient strength, by the *b*-values are defined; I: diffusion and flow-sensitive *b*-values (diffusion gradients); II: diffusion sensitive, flow insensitive *b*-values; III: flow insensitive and noise sensitive *b*-values. The *b*-value independent rician noise level is mentioned as noise floor. *Note*: ADC1 + ADC2 = ADC3. The axis scales, slopes and by this the numeric functions are used as a schematic representation for the general picture and therefore might differ from clinical practice

As observed, ADC-values overlapped between pCR/non-pCR groups, and between studies. This may partly be explained by different *b*-value combinations used for calculating ADC-values [38]. For example, including perfusion-sensitive low *b*-values can overestimate ADC, whereas using (diffusion and noise sensitive) high *b*-values potentially underestimate ADC (Fig. 6, Additional file 1: Figure A and B, illustrating the different slopes). Moreover, the *b*-values can be constructed in different ways (i.e., depending on the DWI gradient properties). Theoretically, diffusion time, and thereby the DWI image, can vary between scans, although the *b*-value is identical. This makes it difficult to compare *b*-values between scanners. Reporting differences in the gradient strength and its timing properties, which may influence measurement results, makes multi-center multi-scanner studies easier to understand. This is important as DWI, by applying a certain *b*-value, can be sensitive to intra- and/or extra-cellular water motion effects (i.e., restriction and hindrance, respectively) and/or perfusion/pseudo-diffusion effects. Additionally, the ADC calculation methods (e.g., the scanner or specific formulas) [39] might not be identical.

The large variation in studies is fairly illustrated by the differences in the baseline mean ADC: in Santamaria et al. and Tozaki et al. between the non-pCR (1.072 × 10^−3^ mm^2^/s vs. 0.64 × 10^−3^ mm^2^/s) and pCR cases (1.025 × 10^−3^ mm^2^/s vs. 0.41 × 10^−3^ mm^2^/s), with *b*-combinations: *b* = 0 s/mm^2^ and *b* = 700 s/mm^2^ or *b* = 50 s/mm^2^ and *b* = 700 s/mm^2^ for Santamaria et al.; and *b* = 500 s/mm^2^ and *b* = 1500 s/mm^2^ for Tozaki et al. [27, 40]. ADC cutoff values for pCR and non-pCR reported in different studies may thus be sensitive to technical heterogeneity. This makes Quality Control (QC) and Quality Assurance (QA), using DWI phantoms [41–43] and patient test–retest procedures [37], essential.

One could argue that, in a longitudinal study, using (flow-sensitive) low *b*-values may have an undesirable effect on the validity of ADC measuring response in highly vascularized tumors. NST reduces vascularization within the ROI and therefore leads to a decrease in the perfusion fraction (*f*), which may cause a decrease in the slope of (a part of) the attenuation curve. Simultaneously the diffusion coefficient increases and compensates this decrease, resulting in a smaller (or even no) difference in ADC between time points. Theoretically, separating the perfusion/pseudo-diffusion and diffusion effects by using > 2 *b*-values and calculating IVIM-parameters could solve this. However, whether this is really beneficial could not be concluded from the included studies in this review due to the small number of studies and heterogeneity. The complexity of choosing the optimal scan-moments and parameters can be observed in Li et al. [44] who suggested that tumors with a relative high ADC during NST are more likely to show pCR, while Tozaki et al. [40] suggested the opposite (Table 7). However, this could not clearly be explained by the DWI acquisition moment during NST.

Besides DWI models [45] and *b*-values, ROI-selection is also crucial for a representative quantitative analysis of each lesion. Using different ROI-definitions (2D/3D) can influence the quantitative results in general, as reported by Bickel et al. [46]. These authors suggested to choose the area of the most aggressive part, the minimum ADC for a 2D-ROI. [46] Other methods are also studied, like whole tumor versus small sub-regions ROI’s [47]. However, these publications are related to lesion classification. It is even more unclear which ROI is most appropriate in a longitudinal setting. Within the ROI, partial volume effects (PVE) might influence (mean) ADC. During therapy, tumor heterogeneity (and thus PVE) may increase and the optimal ROI selection may be affected by various observed shrinkage patterns of breast cancer [24, 48]. Consequently, these aspects make choosing a reliable ROI during and after therapy even more difficult to standardize. Based on systematic review, no optimal ROI technique was identified [49]. In line with the recent study of Wielema et al., regarding the optimal ROI technique for lesion classification using DWI, more extensive research regarding this specific topic in the setting of therapy monitoring is also required.

For identifying the most reliable ROI, in case of small regions of (residual) disease, a sufficient spatial resolution and contrast-to-noise ratio (CNR) between the lesion and the breast parenchyma is required. In DWI, this can be challenging, as often SS-EPI is used with a large field-of-view (FOV) for covering both breasts and thereby compromising spatial resolution due to signal-to-noise ratio (SNR) and scan-time limitations. Therefore, often DCE-images are used as guidance for tumor localization, assisting with identifying lesion(s) at the high *b*-value images. It should be noticed that at higher *b*-values, the SNR decreases and thereby the noise level (rician noise floor) can be reached. To increase SNR for these cases, the number of excitations (NEX) can be increased, which directly will increase the total scan-time. Balancing both (noise ratio and scan-efficiency) can be challenging and will depend on the magnitude of the high *b*-value image. Increasing the highest *b*-value might result in a longer TE, causing a lower SNR, requiring more NEX, and finally a longer acquisition time. Moreover, as there is an inverse relation between image resolution and SNR, recommendations are required discussing the optimal use of DWI for near complete response cases at time-points toward surgery or when small volume lesions (< 1 cm) at baseline are detected (e.g., by using a different or additional high resolution protocol). The development of new DWI sequences addressing this resolution aspect [50] and implementation of post-processing (noise filtering, using advanced DWI models/representations with their considerations [51]) need to be investigated more for these kind of cases. However, it should be noted this would make standardization of DWI for treatment monitoring even more complex.

Analyzing the value of DWI requires measurements coupled to a specific pathological endpoint after NST (pCR/non-pCR). Differences in the histo-pathological analysis (and inter-observer differences in defining the molecular subtype of the diagnostic biopsy [52]) and pCR-definitions can affect this categorization, which further hampers data pooling. Some authors allowed residual DCIS within the group of pCR; others classified it as non-pCR. Furthermore, Liu et al. [16] included Miller & Payne grade 4 (> 90% loss of tumor cells) within the pCR group and Kim et al. [53] labeled those as good responders, whereas others only included grade 5 (no viable tumor cells). Inclusion of DCIS (alone or in combination with grade 4 residual disease) in the pCR group logically leads to different ADC measures than when the pCR group consists of cases without residual DCIS. Noteworthy, while DCIS is not always visible on DWI, because of the spatial resolution, it may still affect ADC-values due to microstructural changes. With the final goal of identifying pCR of the breast after NST in mind, and thereby omitting breast surgery, it seems most appropriate to use a pCR definition of ypT0 (i.e., residual DCIS is not permitted). However, recommendations from the Breast International Group-North American Breast Cancer Group (the BIG-NABCG), on the pathological evaluation of post-NST specimens, still give the option to in- or exclude DCIS from this definition [54, 55]. Aiming at more standardization by making studies more comparable, expert consensus on the most suitable pCR-definition and the definition of radiological complete response on DWI is required.

ADC-values can also vary widely between tumors of different morphological [56] and molecular subtype [57]. Remarkably, in most studies ADC-values were not differentiated by tumor type. Likewise, differences can occur after treatment due to varying NST-regimes. Only four studies reported (absolute/change in) ADC-values for different cancer subtypes, showing differences in distinguishing pCR/non-pCR cases. In other words, all subtypes will likely have specific cutoff values that will also further differ depending on the NST-type. In line with DCE-MRI [7] and PET-CT [58], DWI will likely also have varying diagnostic performance for the response prediction in different subtypes. Partridge et al. [23] and Yuan et al. [22] underlined that also the optimal timing of DWI during NST differ for the molecular subtypes and types of NST. Substantial knowledge about the tumor, its initial and long-term reaction to NST (e.g., cell swelling, apoptosis, and inflammation) is required to determine the optimal timing. Therefore, future DWI research should study identical treatment regimen for specific tumors in large study populations.

Based on this review, identifying pCR seems to be more accurate with parameters that measure differences in ADC-value during NST than with measuring an (absolute) ADC at one or several time point(s). This is likely, because the relative changes (partly) compensate for the variability in the acquisition parameters and biological properties of breast cancers. In general, treatment response is represented by an increase in the lesion’s ADC-value, although even this was not apparent in all studies [59].

Moreover, statistical limitations hamper the potential comparison and pooling of studies. For example, in the QUADAS-2 [15] assessment, risks of bias were observed regarding the research populations. Furthermore, for comparing predictive statistical parameters (PPV/NPV) the prevalence of tumor subtypes needs to be identical. Only a ROC-AUC might give some statistical value to all cases, because it is reported to be prevalence independent [60]. However, as reported in this study, this does not compensate for underlying heterogeneity.

In summary, this review unearths many sources of heterogeneity that are currently present in studies on the use of breast DWI for the prediction of response to NST. This heterogeneity is not limited to acquisitions parameters, but is also caused by large differences in patient populations, biological tumor characteristics, differences in applied therapies, and differences in the used outcome parameters. We acknowledge that besides the factors we specifically addressed even more characteristics in each of these fields could influence DWI measurements. Considering the limited case and study numbers, and all heterogeneity encountered, it would be premature to define the optimal DWI parameters based upon this review. Overall, the level of evidence for response prediction and evaluation using ADC as DWI metric is moderate. However, specific details, such as the influence of the biology of tumors, and the technical aspects of DWI for response prediction only have a low level of evidence [61]. Proper validation aimed at overcoming the translational gaps [62] and, standardization of the study designs (patient inclusion → analysis), requires substantial consensus efforts that are crucial to accelerate optimization, and potential implementation of quantitative-DWI for NST-monitoring in breast cancer patients.

Finally, besides standardization and validation issues, there are also limited data about the cost-effectiveness of MRI in the NST setting [63]. To get an overall idea of the added value of DWI in this NST setting, also cost-effectiveness needs to be analyzed.

By addressing these issues, this review aims to increase awareness on different sources of variability and supplements the works of EUSOBI [14], QIBA [29], Padhani et al. [10] and O’Connor et al. [62], to initiate a future consensus for the use of breast DWI in the treatment monitoring setting.

## Conclusion

Clinical, technical, and epidemiological heterogeneity was observed in all aspects of studies correlating DWI measurements to pCR/non-pCR.

The observed methodological heterogeneity and the small patient numbers make it currently difficult to assess to what extent DWI–MRI might predict pCR. The preliminary conclusion is that the absolute ADC is not (yet) robust for distinguishing pCR/non-pCR, without considering multiple variables. Therefore, multidisciplinary cooperation/consensus is required, to obtain reliable and reproducible longitudinal DWI measurements for identifying non-pCR/pCR cases in specific and well-defined subgroups of patients.

## Supplementary Information

**Additional file 1.** Search term combinations in PubMed.

## Data Availability

Not applicable.

## Abbreviations

ADC: Apparent diffusion coefficient
AUC (ROC): Area under the curve of the receiver operating curve
BIG-NABCG: Breast International Group-North American Breast Cancer Group
CNR: Contrast-to-noise ratio
DCE: Dynamic contrast enhanced
DCIS: Ductal carcinoma in situ
DDC: Distributed diffusion coefficient
DWI: Diffusion-weighted imaging
EUSOBI: European Society of Breast Imaging
FOV: Field-of-view
HER 2: Human epidermal growth factor receptor 2
IVIM: Intravoxel incoherent motion
MeSH: Medical Subject Headings
MRI: Magnetic resonance imaging
NEX: Number of excitations
NPV: Negative predictive value
NST: Neoadjuvant systemic therapy
pCR: Pathologic complete response
PPV: Positive predictive value
PVE: Partial volume effect
QA: Quality assurance
QC: Quality control
QIBA: Quantitative Imaging Biomarkers Alliance
ROI: Region of interest
SEM: Stretched exponential modeling
SNR: Signal-to-noise ratio
SS-EPI: Single-shot echo planar imaging
TE: Echo time
TR: Repetition time
