# Supplementary material for: Factors affecting the value of diffusion-weighted imaging for identifying breast cancer patients with pathological complete response on neoadjuvant systemic therapy: a systematic review
Source: Insights Imaging. 2021 Dec 18;12:187. doi: 10.1186/s13244-021-01123-1 (PMC8684570; doi:10.1186/s13244-021-01123-1)
Supplement: Supplementary file 1 — Additional file 1. Search term combinations in PubMed. [file 13244_2021_1123_MOESM1_ESM.docx]

**ELECTRONIC SUPPLEMENTARY MATERAL**

**(Appendix 1)**

|  |
| --- |
|   Figure A: Schematic overview. Black lines represent the combination of two b-values, resulting in different slopes and different ADC within one lesion. The axis scales, slopes and by this the numeric functions are used as a schematic representation for the general picture and therefore might differ from clinical practice.  Figure B: Zooming in, showing the different slopes in more detail. |

**Search term combinations in Pubmed:**

**Prediction (DCE en DWI)**

**(((((((breast neoplasms[MeSH Terms]) OR (breast tumor OR breast cancer OR breast tumour))) AND (((neo-adjuvant OR neoadjuvant OR neoadjuvant chemotherapy)) OR immunotherapy[MeSH Terms])) OR NAC) AND ((diffusion magnetic resonance imaging[MeSH Terms]) AND (DWI OR DW-MRI OR diffusion OR ADC))) AND ((contrast media[MeSH Terms]) OR (gadolinium OR contrast OR DCE OR multiparametric MRI))) AND (((prognosis[MeSH Terms]) OR predictive value of tests[MeSH Terms]) OR (PPV OR NPV OR imaging biomarker OR prediction OR predictive biomarker OR predictive factor))**

**Evaluation (DCE en DWI)**

**(((((((breast neoplasms[MeSH Terms]) OR (breast tumor OR breast cancer OR breast tumour))) AND (((neo-adjuvant OR neoadjuvant OR neoadjuvant chemotherapy)) OR immunotherapy[MeSH Terms])) AND NAC) AND ((diffusion magnetic resonance imaging[MeSH Terms]) AND (DWI OR DW-MRI OR diffusion OR ADC))) AND ((contrast media[MeSH Terms]) OR (gadolinium OR contrast OR DCE OR multiparametric MRI))) AND ((((treatment outcome[MeSH Terms]) OR tumor burden[MeSH Terms]) OR Response Evaluation Criteria in Solid Tumors[MeSH Terms]) OR (evaluation OR diffusion changes OR therapy respons OR rCR OR pCR OR tumor size))**

**Suggestion in Pubmed:**

**(((((((breast neoplasms[MeSH Terms]) OR (breast tumor OR breast cancer OR breast tumour))) AND (((neo-adjuvant OR neoadjuvant OR neoadjuvant chemotherapy)) OR immunotherapy[MeSH Terms])) AND NAC) AND ((diffusion magnetic resonance imaging[MeSH Terms]) AND (DWI OR dw-mri OR diffusion OR ADC))) AND ((contrast media[MeSH Terms]) OR (gadolinium OR contrast OR DCE OR multiparametric mri))) AND ((((treatment outcome[MeSH Terms]) OR tumor burden[MeSH Terms]) OR Response Evaluation Criteria in Solid Tumors[MeSH Terms]) OR (evaluation OR diffusion changes OR therapy response OR rCR OR pCR OR tumor size))**

**Prediction DWI:**

**((((((breast neoplasms[MeSH Terms]) OR (breast tumor OR breast cancer OR breast tumour))) AND (((neo-adjuvant OR neoadjuvant OR neoadjuvant chemotherapy)) OR immunotherapy[MeSH Terms])) AND NAC) AND ((diffusion magnetic resonance imaging[MeSH Terms]) AND (DWI OR DW-MRI OR diffusion OR ADC))) AND (((prognosis[MeSH Terms]) OR predictive value of tests[MeSH Terms]) OR (PPV OR NPV OR imaging biomarker OR prediction OR predictive biomarker OR predictive factor))**

**Evaluation DWI:**

**((((((breast neoplasms[MeSH Terms]) OR (breast tumor OR breast cancer OR breast tumour))) AND (((neo-adjuvant OR neoadjuvant OR neoadjuvant chemotherapy)) OR immunotherapy[MeSH Terms])) AND NAC) AND ((diffusion magnetic resonance imaging[MeSH Terms]) AND (DWI OR DW-MRI OR diffusion OR ADC))) AND ((((treatment outcome[MeSH Terms]) OR tumor burden[MeSH Terms]) OR Response Evaluation Criteria in Solid Tumors[MeSH Terms]) OR (evaluation OR diffusion changes OR therapy respons OR rCR OR pCR OR tumor size))**

**Suggestion in Pubmed:**

**((((((breast neoplasms[MeSH Terms]) OR (breast tumor OR breast cancer OR breast tumour))) AND (((neo-adjuvant OR neoadjuvant OR neoadjuvant chemotherapy)) OR immunotherapy[MeSH Terms])) AND NAC) AND ((diffusion magnetic resonance imaging[MeSH Terms]) AND (DWI OR dw-mri OR diffusion OR ADC))) AND ((((treatment outcome[MeSH Terms]) OR tumor burden[MeSH Terms]) OR Response Evaluation Criteria in Solid Tumors[MeSH Terms]) OR (evaluation OR diffusion changes OR therapy response OR rCR OR pCR OR tumor size))**
